# DISMIR: Deep learning-based noninvasive cancer detection by integrating DNA sequence and methylation information of individual cell-free DNA reads

**DOI:** 10.1093/bib/bbab250

**Published:** 2021-07-09

**Authors:** Jiaqi Li, Lei Wei, Xianglin Zhang, Wei Zhang, Haochen Wang, Bixi Zhong, Zhen Xie, Hairong Lv, Xiaowo Wang

**Affiliations:** Ministry of Education Key Laboratory of Bioinformatics; Center for Synthetic and Systems Biology; Bioinformatics Division, Beijing National Research Center for Information Science and Technology; Department of Automation, Tsinghua University, Beijing 100084, China; Ministry of Education Key Laboratory of Bioinformatics; Center for Synthetic and Systems Biology; Bioinformatics Division, Beijing National Research Center for Information Science and Technology; Department of Automation, Tsinghua University, Beijing 100084, China; Ministry of Education Key Laboratory of Bioinformatics; Center for Synthetic and Systems Biology; Bioinformatics Division, Beijing National Research Center for Information Science and Technology; Department of Automation, Tsinghua University, Beijing 100084, China; Ministry of Education Key Laboratory of Bioinformatics; Center for Synthetic and Systems Biology; Bioinformatics Division, Beijing National Research Center for Information Science and Technology; Department of Automation, Tsinghua University, Beijing 100084, China; Ministry of Education Key Laboratory of Bioinformatics; Center for Synthetic and Systems Biology; Bioinformatics Division, Beijing National Research Center for Information Science and Technology; Department of Automation, Tsinghua University, Beijing 100084, China; Ministry of Education Key Laboratory of Bioinformatics; Center for Synthetic and Systems Biology; Bioinformatics Division, Beijing National Research Center for Information Science and Technology; Department of Automation, Tsinghua University, Beijing 100084, China; Ministry of Education Key Laboratory of Bioinformatics; Center for Synthetic and Systems Biology; Bioinformatics Division, Beijing National Research Center for Information Science and Technology; Department of Automation, Tsinghua University, Beijing 100084, China; Ministry of Education Key Laboratory of Bioinformatics; Center for Synthetic and Systems Biology; Bioinformatics Division, Beijing National Research Center for Information Science and Technology; Department of Automation, Tsinghua University, Beijing 100084, China; Ministry of Education Key Laboratory of Bioinformatics; Center for Synthetic and Systems Biology; Bioinformatics Division, Beijing National Research Center for Information Science and Technology; Department of Automation, Tsinghua University, Beijing 100084, China

**Keywords:** cell-free DNA, methylation, deep learning, cancer detection, liquid biopsy

## Abstract

Detecting cancer signals in cell-free DNA (cfDNA) high-throughput sequencing data is emerging as a novel noninvasive cancer detection method. Due to the high cost of sequencing, it is crucial to make robust and precise predictions with low-depth cfDNA sequencing data. Here we propose a novel approach named DISMIR, which can provide ultrasensitive and robust cancer detection by integrating DNA sequence and methylation information in plasma cfDNA whole-genome bisulfite sequencing (WGBS) data. DISMIR introduces a new feature termed as ‘switching region’ to define cancer-specific differentially methylated regions, which can enrich the cancer-related signal at read-resolution. DISMIR applies a deep learning model to predict the source of every single read based on its DNA sequence and methylation state and then predicts the risk that the plasma donor is suffering from cancer. DISMIR exhibited high accuracy and robustness on hepatocellular carcinoma detection by plasma cfDNA WGBS data even at ultralow sequencing depths. Further analysis showed that DISMIR tends to be insensitive to alterations of single CpG sites’ methylation states, which suggests DISMIR could resist to technical noise of WGBS. All these results showed DISMIR with the potential to be a precise and robust method for low-cost early cancer detection.

## Introduction

Cell-free DNAs (cfDNA) are degraded DNA fragments released to body fluids such as plasma and urine mainly brought by apoptosis or necrosis cells [1]. It was reported that in the early stage of cancer when there are no significant clinical symptoms on patients, the state of DNA in cancer cells has already changed [2] and can be detected in the plasma of cancer patients as circulating tumor DNA (ctDNA) [3] . With the development of high-throughput sequencing technologies, noninvasive approaches by identifying cancer signals in cfDNA sequencing data are emerging as novel liquid biopsy methods for cancer diagnosis [4].

The majority of cfDNA studies focus on the mutation of oncogenes. The existence and fraction of ctDNA in the total cfDNA is calculated by detecting certain mutations in a small oncogene panel [5, 6]. However, the fraction of ctDNA in early-stage cancer is too low to detect without an ultradeep sequencing method [7, 8]. Besides, mutations that drive carcinogenesis are usually diverse, leading to heterogeneity across different patients or across different loci in tumor tissues, which limits the potent of detecting cancer by ctDNA mutation [9]. Some other studies tried to detect the rearrangement of chromosomes during carcinogenesis by cfDNA such as copy number alterations [10, 11] and fragmentation patterns [12, 13] and found interesting relationships between these signatures and cancer. However, as cfDNA sequencing data are mixed data with low signal-to-noise ratios, these low-resolution signatures can hardly be distinguished from noise when detecting early-stage cancer, therefore cannot be solely applied as accurate biomarkers for early-stage cancer detection.

The methylation states of DNA are altered in the early stage of cancer widespread across the whole genome [14, 15], which warranties methylation as an informative feature for early-stage cancer detection. Therefore, integrations of the methylation states on different CpG sites [16] or in different subgenomic regions [17] are promising approaches to enhance the precision of cancer detection. Furthermore, as the fraction of ctDNA in the total cfDNA was shown to be concordant with tumor burden [18], deconvolution of cfDNA to infer its origin becomes a hopeful approach to estimate the existence and severity of cancer [19]. Though, the performance of such methods is still limited by the low signal-to-noise ratio. Recently, probabilistic methods such as CancerLocator [20] were introduced to predict the location of cancer and tumor burden, which realized promising results on patient plasma samples. An upgraded probabilistic approach-based method called CancerDetector [21] was then proposed and outperformed CancerLocator. CancerDetector predicted the source of cfDNA at the resolution of individual sequencing reads using the local correlation of methylation states between adjacent CpG sites, providing a novel read-based sight to investigate cfDNA sequencing data. However, different depths of sequencing data may introduce systematic deviation to the prediction results of CancerDetector, which could further reduce the accuracy of cancer diagnosis.

Previous work suggested that the methylation states are partly *cis*-regulated by the surrounding DNA sequence [22, 23]. Therefore, the surrounding DNA sequence may provide valuable information to analyze the methylation state and predict the source of individual reads. Here, we adopted a deep learning model named DISMIR to predict the source of individual reads. DISMIR can integrate the DNA sequence and methylation information of the selected differentially methylated regions (DMRs) across the whole genome, and thus enables the prediction accuracy even at very low sequencing depths. Besides, we introduced a new feature termed as ‘switching region’ to find specific DMRs suitable for the source prediction of individual reads to further improve the accuracy. DISMIR successfully achieved an area under the receiver operating characteristic (ROC) curve (AUC) of 0.9969 ± 0.0016 (mean ± SD) in the diagnosis of hepatocellular carcinoma (HCC) with low sequencing depth cfDNA whole-genome bisulfite sequencing (WGBS) data (coverage from 1× to 10×). When subsampling the sequencing data to an ultralow sequencing depth (from 0.01× to 0.1×), DISMIR still achieved an AUC of 0.9112 ± 0.0307. Analysis of the deep learning model showed that DISMIR successfully extracted DNA sequence and methylation patterns related to HCC across the whole genome and was more sensitive to global methylation alterations, which made DISMIR able to resist to technical noise of WGBS. The results suggested DISMIR can do better cancer diagnosis with low sequencing depths at the early stage of cancer by successfully combining the information of DNA sequence and methylation together, which could be of great help to further clinical application.

## Materials and methods

### Overview

**Figure 1:**
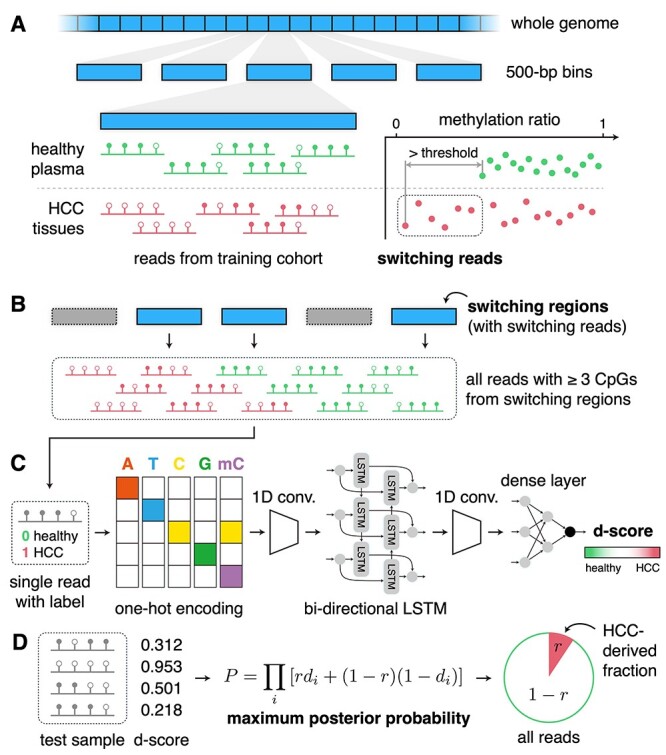
Overview of DISMIR. (**A**) Identifying cancer-specific DMRs across the whole genome with the definition of switching regions and switching reads. (**B**) Collecting all reads with three or more CpG sites from switching regions for further analysis. (**C**) Training a deep learning model to calculate the d-score of each individual read. (**D**) Predicting tumor fraction of a sample by maximizing the posterior probability.

The ultimate goal of DISMIR is to diagnose cancer by integrating DNA sequence and methylation information in plasma cfDNA WGBS data. The diagnosis is performed by predicting the source of each read and then estimating the proportion of tumor-derived reads in the total cfDNA. The overall procedure of DISMIR comprises four main steps: (1) identify the cancer-specific DMRs of cancer tissues in comparison with healthy people’s plasma across the whole genome as candidate biomarkers (Figure 1**A**). (2) Screen out reads in plasma cfDNA WGBS data that are located in the cancer-specific DMRs (Figure 1**B**). (3) Train a deep learning model to integrate DNA sequence and methylation information with these data to mark each read a value named d-score as the potent that the read is derived from cancer tissues (Figure 1**C**). (4) Estimate the fraction of tumor-derived reads of a plasma sample by all d-scores to infer whether the plasma donor is suffering from cancer (Figure 1**D**). Here we adopted HCC as an example to validate the performance of DISMIR.

### Data collection and processing

The data employed in this study contain single-end WGBS data (coverage from 1× to 3×) of plasma cfDNA as well as HCC cancer tissues from European Genome-Phenome Archive database (EGA) with accession number EGAS00001000566 [17] and EGAS00001001219 [24]. The cancer tissue data were from 13 HCC patients. The plasma cfDNA data were from 32 healthy people, 8 hepatitis B virus (HBV) carriers without cancer and 16 HCC patients (to get rid of information leakage, plasma cfDNA data, which have paired cancer tissues involved in this study were excluded). Besides, paired-end WGBS data (coverage around 10×) of plasma cfDNA from four healthy people and four HCC patients with EGA accession number EGAS00001002728 [21] were also used to test the performance of DISMIR.

The training cohort contains nine HCC patients’ cancer tissues WGBS data and 18 randomly chosen healthy people’s plasma WGBS data, which were randomly chosen from the dataset EGAS00001000566 for 10 times. WGBS data of the remaining 18 healthy people’s plasma, 8 HBV carriers’ plasma and 20 unpaired HCC patients’ plasma compose the test cohort. The WGBS data of the remained four HCC tissues were used for simulation experiments to evaluate the effect of our approach. Details of the training and test cohort composition were shown in Supplemental Table 1.

We used BS-Seeker2 [25] to align all these WGBS data to hg19, removed PCR duplicates and then called the methylation states of all CpG sites for subsequent analysis.

### Identifying HCC-specific DMRs

Identifying HCC-specific DMRs across the whole genome could provide valid cancer-related information refraining from the unconcerned variation of methylation states among different samples. As the sequencing data of plasma cfDNA could be regarded as a mixed signal of tumor-derived cfDNA and basal cfDNA, which is similar to cfDNA at the healthy state, we should use the reads from regions where the methylation patterns are different between cancer tissues and healthy plasma cfDNA. Previous studies [26–30] have produced many methods to define DMRs. These methods mainly focused on the statistics of total reads from a certain genome region. However, fractions of tumor-derived reads in plasma cfDNA are usually ultralow especially at the early stage. The identification of tumor-derived reads would be greatly dampened by outliers from healthy tissues in the calculation of traditional statistics. Therefore, we defined DMRs as regions where the methylation patterns of tumor-derived reads are distinguishable from patterns of reads from healthy plasma to enhance the cancer-related signal at read-resolution in cfDNA sequencing data.

Based on such assumption, we introduced a new feature named ‘switching regions’ and ‘switching reads’, which were defined with the following steps (Figure 1**A**). Firstly, we divided the whole genome into 500-bp regions without overlaps and filtered out regions with <25 reads in all training-cohort samples. Then we calculated the methylation ratios of all DNA fragments from a certain region to get their distributions in cancer tissues as well as cfDNA from healthy plasma. Here we only used reads with three or more CpG sites. Next, we compared the maximum and minimum values of two distributions. For instance, to identify hypomethylated switching regions, we denoted the healthy plasma’s minimum methylation rate of all reads in a region as *H*_min_ and denoted the cancer tissues’ minimum methylation rate of all reads in this region as *T*_min_. When *H*_min_ − *T*_min_ is larger than a certain threshold, this region is defined as a switching region. All reads from switching regions with methylation rates lower than *H*_min_ are defined as switching reads. The hypermethylated switching regions were defined in a similar way. As HCC shows a significant genome-wide hypomethylation pattern in comparison with healthy tissues [17], here we focused on the hypomethylated switching regions in HCC.

The value of the threshold determines how many switching regions are identified. When the threshold is higher, fewer regions will be identified as switching regions, which may cause the shrink of reads numbers and thus result in the reduction of precision. On the contrary, lower thresholds lead to more switching regions, which consumes more time for deep model training. Here we observed the relationship between the threshold and the amount of hypomethylated switching regions as well as the relationship between the threshold and the accuracy of the model (Supplemental Figure 1) and set the threshold as 0.3 to ensure that the coverage of selected hypomethylated switching regions (mean number of regions: 3130.5, mean coverage: 1.565 Mb) is similar to that of CancerDetector DMRs (mean coverage: 1.515 Mb).

### Predicting the source of each read with a deep learning model

To gain a valid and comprehensive model to depict the DNA sequence and methylation pattern in tumor-derived reads in cfDNA WGBS data, we built a deep learning model to predict the potent that a read is derived from cancer tissues, termed as d-score. All reads with three or more CpG sites from switching regions were used to train the deep learning model. By attaching label to each read according to its source (from healthy plasma as 0, from cancer tissues as 1), we converted this problem into a binary classification problem of reads. For each read, the first 5 bp at the 5′ end was trimmed to avoid the influence of adapters. Then all reads were trimmed at the 3′ end to a same length (*L* = 66 in this study) to unify the input format. We randomly subsampled the reads to ensure the balance between the amount of two sample types and reserved 20% of these reads for kernel visualization.

Here we referred to the structure of DanQ model [31], which was built to quantify the function of DNA sequences, and made some adjustments on it to serve as the core of the deep learning model (Figure 1**C**). Each base of a unified read was encoded into a one-hot matrix according to the nucleobase, and the methylation state of the base was also encoded, where 1 presents methylated and 0 presents unmethylated. Therefore, each input read was encoded into a *L* × 5 matrix. After the input layer, we sequentially added a one-dimensional (1D) convolution layer, a maxpooling layer, a bi-directional LSTM layer, a 1D convolution layer, a flatten layer and three dense layers. The output of the model was a continuous value denoted as d-score between 0 and 1 corresponding to the label of each read. The closer the d-score is to 1, the more likely the read is from a cancer tissue. Details of the deep learning model were shown in Supplemental Figure 2.

### Estimating the fraction of tumor-derived cfDNA

The d-score calculated by the deep learning model was treated as the probability that the read is from a cancer tissue. For a tested sample with *n* reads and their d-scores *d*_1_, *d*_2_, … *d_n_* (Figure 1**D**), we inferred the proportion of reads from cancer tissue according to these d-scores by calculating the maximum posterior probability inspired by CancerDetector [21]. When given the ratio of tumor-derived reads as *r* for a sample and assuming that d-scores of each read are independent, we could get the posterior probability of this sample with these d-scores as *P*:

$$ P=\prod_{i=1}^n\left[r\times{d}_i+\left(1-r\right)\times \left(1-{d}_i\right)\right] $$

When maximizing *P*, we could get the estimated ratio of tumor-derived reads by DISMIR denoted as $\hat{r}$, which could be regarded as the risk that the plasma donor is suffering from cancer for cancer diagnosis:

$$ \hat{r}=\underset{r\in \left[0,1\right]}{\mathrm{argmax}}\prod_{i=1}^n\left[r\times{d}_i+\left(1-r\right)\times \left(1-{d}_i\right)\right] $$

To calculate the maximum posterior probability, we first calculated *P* with every possible *r* from 0 to 1 with a step equal to 0.001 and then found the *r*, which led to the maximum value of *P*.

### Visualizing kernels of the deep learning model by position frequency matrices (PFM)

We visualized kernels of the deep learning model to figure out the DNA sequence and methylation patterns that the model focused on. After finishing the training of the deep learning model, we took out the weight matrices of all kernels in the first 1D convolution layer. We then used the reserved 20% reads in the training set on every possible position as inputs and calculated their activation values by the weight matrices. For each weight matrix, we located the top 1% output values and got the corresponding inputs. These inputs were superposed together with their activation values as weights to calculate the frequency of each base and the methylation state as PFMs.

## Results

### DISMIR achieved high precision in early-stage HCC detection

We identified switching regions and trained DISMIR on a randomly selected training cohort. Then, we tested DISMIR on remained samples as the test cohort for 10 times (see Materials and Methods for details). For every random selection, we trained DISMIR for 10 times with the same data and then applied the model on the test cohort. The average d-score of each individual read was calculated as the final d-score for downstream estimation of tumor fraction to get rid of the randomness of the deep learning method.

**Figure 2:**
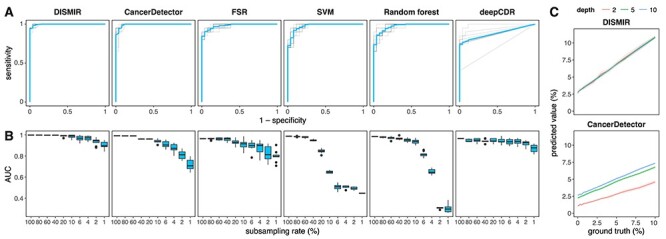
Results of DISMIR and other methods on HCC diagnosis. (**A**) ROC curves of different HCC diagnosis methods in the test cohort. Blue lines show the average of the ROC curve. Each method was performed for 10 times with random partition of training and test samples. (**B**) AUCs of different HCC diagnosis methods at different subsampling rates. Each condition was performed for 10 times with randomly subsampling in the test cohort. (**C**) Simulation results at different depths with DISMIR (top) and CancerDetector (bottom). Each condition was performed for 10 times with randomly sampling and mixing. For each graph, CancerDetector denotes the method following the principle of the CancerDetector paper; deepCDR denotes the deep learning model with the same structure as DISMIR trained with reads from the CancerDetector-identified DMRs.

We adopted the receiver operating characteristic (ROC) curve to evaluate the ability of the tumor fraction predicted by DISMIR for distinguishing HCC patients from healthy people. As shown in Figure 2**A**, the AUC of DISMIR was 0.9969 ± 0.0016 (mean ± SD). At the specificity of 100%, DISMIR achieved a sensitivity of 93.94 ± 3.15%; at the sensitivity of 100%, the specificity of DISMIR was 93.46 ± 4.07%.

We further compared our approach with CancerDetector [21]. Following the principle described in CancerDetector (CancerDetector in short), we first detected DMRs from the CpG clusters defined in the CancerDetector paper and then trained the probabilistic model of CancerDetector with these DMRs. The data used in DMR detection and model training contain methylation data measured by the Infinium HumanMethylation450 microarray derived from The Cancer Genome Atlas [32], which are the same as what the CancerDetector paper used, and WGBS data of healthy people’s plasma in our training cohort. Then, we employed the model to perform cancer detection using reads from these CancerDetector-identified DMRs on the test cohort. We found that CancerDetector achieved an AUC of 0.9925 ± 0.0050 (Figure 2**A**). At the specificity of 100%, the sensitivity was 86.50 ± 10.81%. At the sensitivity of 100%, the specificity was 91.15% ± 2.60%. Though the performance reported here is slightly different from the results reported by the original paper of CancerDetector because the training/test sets used in the two studies are not exactly the same, these results suggested that the performance of DISMIR is comparable with CancerDetector.

We then tested whether the predicted values of DISMIR could be used to predict tumor burdens. As shown in Supplemental Figure 3**A**, the estimated ratio of tumor-derived reads ($\hat{r}$) showed a significant correlation between the tumor size (Pearson’s *r* = 0.882, *P*-value = 6.68 × 10^−5^), which is less than the result of CancerDetector (Supplemental Figure 3**B**, Pearson’s *r* = 0.978, *P*-value = 7.91 × 10^−9^). When removing samples with tumor size >6 cm, the correlation was not significant (Pearson’s *r* = 0.168, *P*-value = 0.642), but CancerDetector still showed a significant correlation in this condition (Pearson’s *r* = 0.717, *P*-value = 0.020). The results suggested that though DISMIR could identify patients with small tumors accurately, the predicted value of DISMIR is not effective as the CancerDetector score for predicting small tumor burdens. Actually, as DISMIR was developed to focus on the binary classification problem of cancer patients and healthy people, the prediction values of DISMIR may not be very suitable for the prediction of small tumor size.

We also investigated the result of DISMIR trained with the hypermethylated switching regions. Here we chose one random separation of the training and test cohort and set the threshold as 0.5 for selecting hypermethylated switching regions (number of regions: 3395, coverage: 1.698 Mb). As shown in Supplemental Figure 4**A**, the AUC of DISMIR employing the reads of hypermethylated switching regions was 0.8885, suggesting that the hypermethylated switching regions also contain valuable information for cancer detection. However, this AUC is much lower than the predicted results with hypomethylated switching regions. We further investigated the predicted values of DISMIR using hypo- and hypermethylated switching regions (Supplemental Figure 4**B** and **C**) and found that the hypomethylated switching regions were sufficient for detecting HCC.

To assess the contribution of the deep learning model, we adopted the fraction of switching reads (FSR in short) and trained two traditional machine learning models, SVM and random forests, based on the methylation ratios of each switching region to diagnose HCC. As shown in Figure 2**A**, these methods showed moderate classification accuracies but could hardly serve as effective HCC diagnosis markers in comparison with DISMIR. Besides, we used reads from the CancerDetector-identified DMRs, which had a similar coverage on genome with DMRs defined by switching regions (Supplemental Figure 1**A**), to train the deep learning model of DISMIR (deepCDR in short) and found a lower precision than DISMIR (Figure 2**A**), further advocating the advantage of defining DMRs by switching regions. As a result, both the deep learning model and the definition of switching regions contributed to the great performance of DISMIR.

### Subsampling and simulation results showed DISMIR as an ultrasensitive and robust HCC detection method

To evaluate the performance of DISMIR at low sequencing depths, we randomly subsampled data in the test cohort for 10 times and applied DISMIR and other abovementioned methods on these data. As shown in Figure 2**B**, DISMIR kept high precisions, whereas the accuracy of CancerDetector decreased significantly with the reduction of sequencing depths. When the data were subsampled with a ratio of 1% (coverage from 0.01× to 0.1×), DISMIR still achieved an AUC of 0.9112 ± 0.0307, which is significantly higher than the AUC of CancerDetector (0.7432 ± 0.0463, Mann–Whitney U test, *P*-value = 1.083 × 10^−5^). Interestingly, FSR exhibited higher AUCs at low sequencing depths than CancerDetector, suggesting that defining DMRs by switching regions could resist to noise better than traditional methods. What’s more, deepCDR also showed better performance at low sequencing depths than CancerDetector, which demonstrated the benefit of employing the deep learning model. Meanwhile, accuracies of the two traditional machine learning methods decreased rapidly with the sequencing depth reduction and even lost the discrimination ability when the subsampling ratio was <4% (Figure 2**B**). All the results suggested that learning the joint patterns of DNA sequence and methylation of reads from switching regions by the deep learning model could predict the source of reads more precisely and thus guarantees the sensitivity of HCC diagnosis at ultralow sequencing depths.

We further conducted a simulated dataset to validate the robustness of DISMIR. We randomly sampled reads from WGBS data of HCC tissues and healthy plasma cfDNA, respectively, and mixed them together with different proportions to imitate certain tumor fractions. Besides, the total amount of reads also varied to simulate different sequencing depths. The sampling procedure was repeated for 10 times for each condition. We then tested DISMIR and CancerDetector on the simulated dataset. As shown in Figure 2**C**, the predicted tumor fractions of DISMIR were consistent at different sequencing depths, but those of CancerDetector increased significantly with the increase of sequencing depth, which may introduce bias into the HCC diagnosis approach as the sequencing depths can hardly be exactly the same without a loss-of-information subsampling procedure. The results suggested that DISMIR is highly robust at different sequencing depths and thus is more applicable.

### Kernels of DISMIR paid attention to joint patterns of DNA sequence and methylation

**Figure 3:**
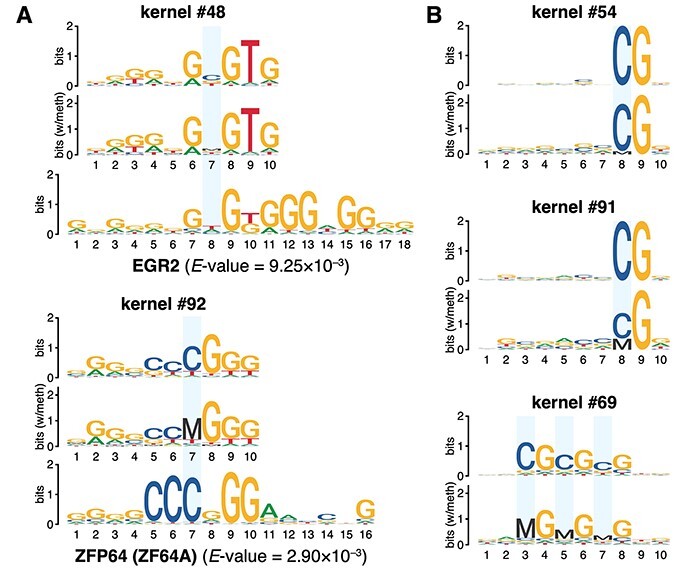
Visualized convolution kernels of the deep learning model. (**A**) Visualized kernels matched to known HCC-related motifs. (**B**) Visualized kernels focusing on the methylation state of CpG sites. Cytosines at CpG sites are marked with blue rectangles.

To investigate how DISMIR distinguished HCC-derived cfDNA fragments from others by employing DNA sequence and methylation information, we tried to interpret the deep learning model of DISMIR by investigating the network details. We visualized the kernels of the first 1D convolution layer by calculating their PFMs (see Materials and Methods for details). We compared the sequence patterns of these PFMs with known motifs by TOMTOM [33] and merged the *E*-values assigned by TOMTOM from 10 times of training with Fisher’s combined probability test. A total of 28 motifs were identified as significant motifs (*P*-value <0.05) matching with the kernel PFMs (Supplemental Table 2). Interestingly, many of these motifs were related to HCC (Supplemental Table 2). For example, as shown in Figure 3**A**, two kernels were matched to the EGR2 and ZFP64 (ZF64A) motif, respectively. EGR2 is an antitumor transcriptional factor, the induction of which could suppress the malignancy of HCC [34, 35]. Meanwhile, the expression of ZFP64 was shown to be positively correlated to the overall survival of advanced HCC patients with the treatment of a second-line therapy [36].

We further visualized the kernels with the methylation information. We treated the methylated cytosine (noted as ‘M’) and the unmethylated cytosine (noted as ‘C’) as two different base and then visualized the kernels in five-base logos. The results were similar with four-base logos except for significant difference at CpG sites. By such visualization, we successfully found the evidence that the deep learning model combined sequence with methylation information together. As shown in Figure 3**A**, the cytosine at the CpG site of the ZFP64-like kernel was almost fully methylated, suggesting the methylation state on this motif was highly coordinated with its flanking DNA sequence pattern during HCC detection. What’s more, we also found several kernels concentrated to different methylation states of CpG sites at different positions of reads (Figure 3**B**). For example, both kernel #54 and #91 paid attention to the CpG site at the 8th position, but they attached quite different importance to the methylation state of the CpG site (Figure 3**B**). Therefore, with other kernels that might pay more attention to the information of joint patterns of DNA sequence and interior methylation, the deep network could thus combine the information together as the preliminary pattern extraction of a whole read for further analysis to predict the source of the read more accurately.

### DISMIR employed the joint pattern of DNA sequence and methylation to distinguish HCC-derived reads

**Figure 4:**
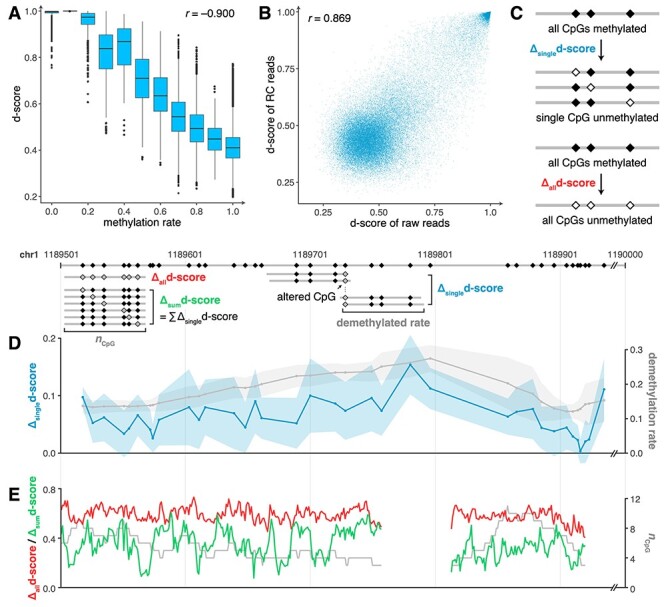
The joint pattern of DNA sequence and methylation decides the prediction of DISMIR. (**A**) The relationship between methylation rates and d-scores of all reads. (**B**) The relationship of d-scores of raw reads and their RC reads. (**C**) The schematic diagram depicting how reads with altered methylation states were generated for downstream analysis. (**D**) Δsingled-score (blue line) and the corresponding demethylation rate (gray line) at different positions in the selected switching region. Colored shadows show the standard deviation of Δsingled-score. (**E**) Δalld-score (red line), Δsumd-score (green line) and the corresponding CpG count (gray line) at different positions in the selected switching region.

Though kernel visualization suggested both DNA sequence and methylation information were processed in DISMIR, less was known about whether DNA sequence and methylation decided the results jointly. Therefore, we investigated the relationship between the methylation ratios of all reads and their d-scores derived by DISMIR (Figure 4**A**), which showed a significant negative correlation (Pearson’s *r* = −0.900). However, d-scores of reads with similar methylation ratios varied enormously. If the score of each read was assigned by its methylation ratio, the correlation should be much higher. Besides, as the methylation states of CpG dyads on both strands are correlated but could be different in some conditions [37], we generated reverse complementary (RC) reads of raw reads from cancer tissues that were not in the training set with the same methylation state at every CpG dyad; thus, the paired raw read and RC read shared the same methylation ratio. We then used DISMIR to predict the d-scores of raw reads and RC reads (Figure 4**B**). The results suggested that DISMIR successfully found the correlated pattern of reads derived from different strands (Pearson’s *r* = 0.869), while there exhibited some difference between them. All the results showed that DISMIR determined the d-score of a read by more beyond its methylation ratio.

**Figure 5:**
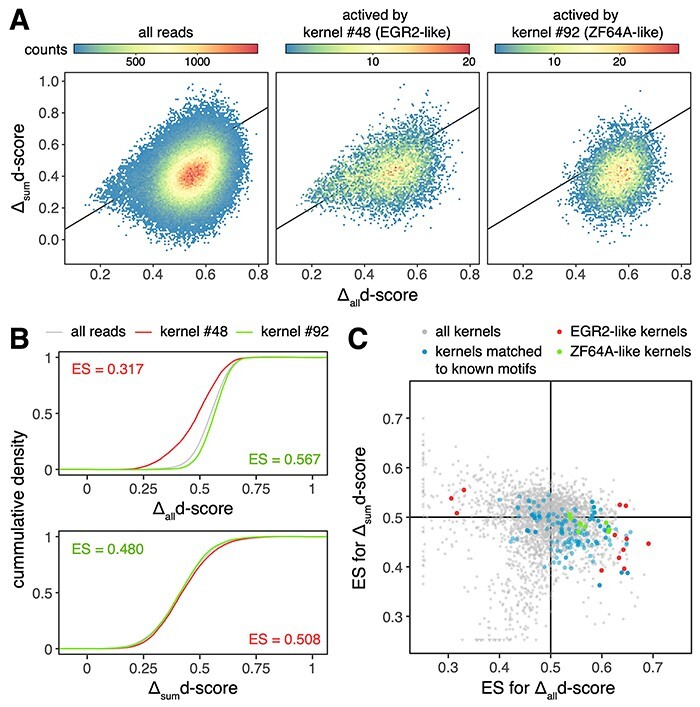
DISMIR and its kernels can resist to methylation state alterations of single CpG sites. (**A**) The distribution of Δalld-scores and Δsumd-scores of all reads (left) and certain kernel-activated reads (middle and right). The black lines are diagonal lines where Δalld-scores are equal to Δsumd-scores. (**B**) The cumulative distribution function of Δalld-scores and Δsumd-scores of reads shown in (**A**). ES of the Mann–Whitney U test are shown. (**C**) The distribution of ES of the Mann–Whitney U test for Δalld-scores and Δsumd-scores of reads activated by certain kernels in comparison with them of all reads, which was performed the same as shown in (**B**). For (**A**) and (**B**), results of reads with three CpG sites were shown. For (**C**), results of reads with three, four and five CpG sites were shown with performing model training for 10 times. Black lines show where ES equals to 0.5 and divide the plane into four quadrants.

Thus, we investigated the collaboration of DNA sequence and methylation within switching regions. Here we gave a sample from a certain switching region with a length of 500 bp on chromosome 1. We generated all possible reads with the same length as the input reads with three or more CpG sites within the region. All CpG sites on these reads were set to be methylated, and their d-scores were calculated by DISMIR. Then, the methylation state of each single CpG site on all reads was altered to be unmethylated. The d-scores changed correspondingly with a magnitude denoted as Δ_single_d-score (Figure 4**C**). Similarly, we examined reads with all CpG sites altered to the unmethylated state and denoted the change of d-score as Δ_all_d-score (Figure 4**C**). Interestingly, alterations of methylation states on different CpG sites contributed differently to the change of d-scores (Figure 4**D**), which couldn’t be fully explained by the alteration of methylation ratios. Besides, when we altered reads from a whole methylated to a whole unmethylated state, though with the same alteration of methylation ratios, changes of d-scores varied across the region (Figure 4**E**), which was not entirely determined by the amount of CpG sites on reads. All the results showed that DISMIR assigned different importance to different CpG sites according to their surrounding DNA sequences. We further added all Δ_single_d-scores of a read together as Δ_sum_d-score and found that almost all Δ_sum_d-scores were less than the corresponding Δ_all_d-scores (Figure 4**E**). The results suggested that DISMIR may focus more on the global methylation alteration rather than just gather the impact of each single CpG site’s methylation alteration together.

### Motif-related kernels of DISMIR could resist to methylation state alterations of single CpG sites

As shown in Figure 4**F**, the d-score change with the alteration of all CpG sites on a read was much higher than the sum of d-score changes with alterations of each single CpG site. This result hinted that DISMIR might pay more attention to global methylation state alterations, which are familiar in cancer tissues. By contrast, alterations of single CpG sites are usually confounded by technical noise during WGBS, thus should be considered with smaller weights when discriminating the origin of reads. To further investigate whether DISMIR could resist to methylation state alterations of single CpG sites, we considered all possible reads with more than three CpG sites in all switching regions and calculated their Δ_all_d-scores and Δ_sum_d-scores. To avoid the confounding of the counts of CpG sites, we grouped the reads by the CpG count and analyzed each group, respectively. Interestingly, DISMIR paid more attention to global alteration of CpG states beyond the additive model of single alterations (the left panel of Figure 5**A** showed results of all reads with three CpG sites; reads with more CpG sites showed similar patterns). The results suggested DISMIR worked as a filter against the influence of the methylation alterations on single CpG sites, which have low signal-to-noise ratios in comparison with global methylation alterations.

We then analyzed the relationship between d-score changes and DISMIR kernels. We calculated the activation values of each kernel on every possible read with all CpG sites set as methylated and reads with the top 0.1% highest activation values were regarded as reads that could highly activate this kernel. Interestingly, the distribution of d-score changes of kernel-activated reads tended to be different from the distribution of all reads (Figure 5**A** and **B**). We further compared the distribution of Δ_all_d-scores and Δ_sum_d-scores of these kernel-activated reads and all reads with the Mann–Whitney U test. As sample sizes of both distributions were huge, the *P*-value of the test was overpowered for subtle difference. Therefore, we adopted the AUC statistic between two distributions, which could be directly derived from the Mann–Whitney U test [38], as the effect size (ES) of the test to quantify the difference between two distributions. As shown in Figure 5**C**, some kernels tended to filter out the influence of single-site demethylation but could focus on whole demethylation (located in the 4th quadrant of Figure 5**C**). We further investigated kernels matched to known motifs (blue points in Figure 5**C**) and found that these kernels are more likely to be located in the 4th quadrant (odds ratio = 4.158, Fisher’s exact test *P*-value = 4.59 × 10^−23^). As the randomness of the training of the deep learning model, kernels may differ across different training, but functional kernels that are more likely to be assigned to known motifs emerged repeatedly in different trainings. These functional kernels, as shown in Figure 5**C**, could resist to the demethylation of single CpG sites, ensuring the high robustness of DISMIR.

## Discussion

In this study, we developed a deep learning-based approach called DISMIR to predict whether reads in plasma cfDNA WGBS data are derived from tumor and further adopted the predicted fraction of tumor-derived reads to diagnose cancer. DISMIR achieved outperformed results in HCC detection, especially at low sequencing depths, which makes it possible to be a low-cost cancer-detection method. The predicted fractions of tumor-derived reads are also stable at different sequencing depths, so that we can assign a unified threshold to samples with various sequencing depths for cancer diagnosis. These advantages make DISMIR more likely to be applied in clinical practice.

The outperformance of DISMIR was mainly contributed by the novel design of the deep learning model. We built a deep network to combine the DNA sequence and methylation information together for each read. Therefore, DISMIR could grasp sequence motifs related to cancer and extract the joint patterns of DNA sequence and methylation across different regions from the whole genome to ensure the source prediction of individual reads more accurate. As a contrast, methods with only methylation information such as SVM, random forests and FSR performed much worse than DISMIR (Figure 2), showing the advantage of integrating the information of DNA sequence and methylation. Besides, information derived from different regions makes the model more robust and thus guarantees the precision of prediction even at extremely low sequencing depths.

Deep learning approaches usually require a large number of samples for training. However, the difficulty of obtaining clinical samples and the expensive cost of WGBS experiments limit the sample size of cfDNA WGBS data. Thus, in this study, we regarded each individual read from the switching regions as a sample instead of the statistics of all reads from a DMR. The deep learning model didn’t learn the pattern of sequencing samples as a whole but all sequencing reads as individuals. Therefore, the amount of the individual reads is large enough to meet the requirement of deep learning.

Different to the definitions of DMRs in other approaches, here we introduced a novel method to identify DMRs called switching regions to enrich reads with more distinguishable methylation patterns. As previous studies suggested, methylation patterns at the resolution of read level could make the model more sensitive [39]. In comparison with traditional definition of DMRs, switching regions are more sensitive to evade outlier reads, which might introduce significant noise to the read-resolution deep learning model. DNA fragments from switching regions contain more specific features and could thus enhance the precision of signal detection resisting to noise at low sequencing depths. Thus, defining DMRs by switching regions is more suitable for models employing individual reads as inputs. Furthermore, kernels of DISMIR that were related to known motifs paid more attention to global alteration of methylation states but less attention to methylation state alterations of single CpG sites, which made DISMIR able to resist to technical noise of WGBS and thus enhanced the robustness of DISMIR.

We found that several motifs that kernels of the deep learning model focused on were related to cancer, which showed the powerful capacity of feature extraction as well as good interpretability of the deep learning model. Furthermore, some kernels that didn’t match with known cancer motifs may contain novel information related to cancer, especially in the process of epigenomic regulation. The deep learning method, which integrates DNA sequence and methylation information together, also provides a data-driven method for us to unveil the interaction between genomes and epigenomes [40, 41]. In addition, the deep learning-based method can also be applied in other multimodal data to extract useful joint patterns to find out new rules in certain biological processes.

DISMIR could be easily applied on the detection of other cancer types. As model accuracy and training efficiency shows a trade-off determined by the threshold of switching regions, we suggest users to pretrain the model with training data or simulated data to find a computation-acceptable threshold with high accuracies. Besides, as the hyper- and hypomethylation profiles differ among cancer types, we suggest users to train two DISMIR models using the hyper- and hypomethylated switching regions, respectively, and then select or integrate the outputs of two models to determine the risk of cancer.

This study can be further improved in several ways. Firstly, more cfDNA samples could be involved in the testing cohort to further evaluate the precision of the method. Besides, as the tumor samples used for model training may not be just composed of cancer cells [42], we can develop correction methods based on the tumor purity before model training to get more accurate predictions of tumor-derived cfDNA. Furthermore, though this study employed HCC to evaluate the performance, the method could be used and should be validated on more kinds of cancers. In addition, new approaches based on this method could be further developed to transfer the features learnt from one kind of cancer to the model of another cancer and thus realize efficient pan-cancer detection.

**Key points**

- DISMIR exhibits high accuracy and robustness in the detection of cancer with WGBS data even at ultralow sequencing depths. The results demonstrated that DISMIR achieved an AUC of 0.9112 ± 0.0307 at ultralow depths from 0.01× to 0.1× in the diagnosis of early-stage HCC.
- DISMIR is a deep learning-based method that integrates the information of DNA sequence and methylation of each read, which was proved to be able to resist to technical noise. This framework could be used to discover the interaction between genomes and epigenomes.
- DISMIR predicts the source of each read and then estimates the cancer risk according to the prediction results of all reads, which is highly suitable for samples that are mixtures of signals such as cfDNA. DISMIR introduces ‘switching region’ to define cancer-specific differentially methylated regions, which can enrich the cancer-related signal at read-resolution.
- DISMIR can serve as a precise and robust noninvasive detection method for various types of cancers at the early stage. DISMIR requires lower sequencing depths than other methods and thus is more likely to be applied in clinical practice.

## Supplementary Material

DISMIR_Supplementary_Materials_R2_bbab250

## Data Availability

The source code of DISMIR is available from GitHub (https://github.com/XWangLabTHU/DISMIR). All WGBS data utilized in this study are from European Genome-Phenome Archive database (EGA) with the accession number EGAS00001000566 [17], EGAS00001001219 [24] and EGAS00001002728 [21].

## Notes

**Jiaqi Li** is a PhD candidate at the Department of Automation, Tsinghua University. He is mainly engaged in machine learning, deep learning and the applications of artificial intelligence in bioinformatics.

**Lei Wei** earned his PhD degree at Tsinghua University. He is now a postdoc at the Department of Automation, Tsinghua University. His research interests include machine learning, systems biology and synthetic biology.

**Xianglin Zhang** is a PhD candidate at the Department of Automation, Tsinghua University. His research focuses on the development of pattern recognition algorithms for high-throughput sequencing data.

**Wei Zhang** is a PhD candidate at the Department of Automation, Tsinghua University. His research focuses on the development of pattern recognition algorithms for high-throughput sequencing data.

**Haochen Wang** is a PhD candidate at the Department of Automation, Tsinghua University. His research focuses on the design of synthetic biological components based on artificial intelligence.

**Bixi Zhong** is a master student at the Department of Automation, Tsinghua University. Her research focuses on the deconvolution of high-throughput sequencing data and cancer diagnosis.

**Zhen Xie** is an associate professor at the Department of Automation, Tsinghua University. His research interests include the design and control of artificial molecular machines, synthetic biology, tumor genomics and cell therapy.

**Hairong Lv** is a research associate professor at the Department of Automation, Tsinghua University. His research focuses on the application of artificial intelligence and big data technology in medical and health fields.

**Xiaowo Wang** is a professor at the Department of Automation, Tsinghua University. His research interests include machine learning, bioinformatics, systems biology and synthetic biology.
