# Supplementary material for: DISMIR: Deep learning-based noninvasive cancer detection by integrating DNA sequence and methylation information of individual cell-free DNA reads
Source: Brief Bioinform. 2021 Jul 9;22(6):bbab250. doi: 10.1093/bib/bbab250 (PMC8575022; doi:10.1093/bib/bbab250)
Supplement: DISMIR_Supplementary_Materials_R2_bbab250 [file dismir_supplementary_materials_r2_bbab250.docx]

**SUPPLEMENTAL MATERIALS**

**Supplemental Table 1**. Information of samples and the training / test cohort.

| Dataset | Cancer tissues | cfDNA | | |
| --- | --- | --- | --- | --- |
|  |  | HCC | Healthy | HBV |
| EGAS00001000566 | 13 (A) | 13 (B) | 32 (C) | 8 (D) |
| EGAS00001001219 |  | 3 (E) |  |  |
| EGAS00001002728 |  | 4 (F) | 4 (G) |  |

| Training cohort | Cancer tissues | - Choosing 9 from (A) |
| --- | --- | --- |
|  | Healthy cfDNA | - Randomly choosing 18 from (C) |
| Test cohort | HCC cfDNA | - All samples in (B), (E) & (F) |
|  | Non-cancer cfDNA | - Remaining samples in (C) - All samples in (D) & (G) |
| Simulation experiment | Cancer tissues | - Remaining samples in (A) |
|  | Healthy cfDNA | - Remaining samples in (C) |

**Supplemental Table 2**. Significant motifs (*p*-value < 0.05) matched with the kernel PFMs. *E*-values assigned by TOMTOM from ten times of training were merged with Fisher’s combined probability test and the *p*-values were shown. References of studies that provided the evidence between the motif and HCC were supplemented.

| **Motif** | ***p*-value** | **References** |
| --- | --- | --- |
| ZBT17 | 2.446×10^–7^ |  |
| SP2 | 4.516×10^–6^ | (Zhu *et al.*, 2020) |
| SP3 | 2.276×10^–5^ | (Huang *et al.*, 2015) |
| EGR2 | 7.754×10^–5^ | (Zeng *et al.*, 2017; Wang *et al.*, 2020) |
| ZFX | 1.549×10^–4^ | (Ding *et al.*, 2018) |
| MXI1 | 1.699×10^–4^ | (Sharma *et al.*, 2016) |
| ZF64A | 3.215×10^–4^ | (Bitzer *et al.*, 2016) |
| VEZF1 | 7.591×10^–4^ |  |
| ZN341 | 9.327×10^–4^ |  |
| GATA1 | 1.242×10^–3^ | (Andrieux *et al.*, 2007) |
| MAZ | 1.670×10^–3^ | (Luo *et al.*, 2016) |
| ZN263 | 2.541×10^–3^ |  |
| PATZ1 | 2.758×10^–3^ | (Valentino *et al.*, 2013) |
| RARA | 3.501×10^–3^ | (Sano *et al.*, 2003) |
| **Motif** | ***p*-value** | **References** |
| WT1 | 4.346×10^–3^ | (Mžik *et al.*, 2016) |
| KLF16 | 5.402×10^–3^ |  |
| RARG | 5.627×10^–3^ | (Gan *et al.*, 2016) |
| SP1 | 6.093×10^–3^ | (X. Zhang *et al.*, 2018) |
| PLAG1 | 1.002×10^–2^ | (Cao *et al.*, 2020) |
| EGR1 | 1.020×10^–2^ | (Q. Zhang *et al.*, 2018) |
| TAL1 | 1.298×10^–2^ | (Zou *et al.*, 2019) |
| E2F4 | 1.476×10^–2^ | (Yang *et al.*, 2019, 2) |
| ZN467 | 1.794×10^–2^ |  |
| TBX15 | 2.255×10^–2^ | (Zheng *et al.*, 2016) |
| SMAD3 | 3.128×10^–2^ | (Fu *et al.*, 2018) |
| KLF6 | 3.909×10^–2^ | (Kremer-Tal *et al.*, 2004) |
| ZN770 | 4.691×10^–2^ |  |
| HINFP | 4.705×10^–2^ | (Zhu *et al.*, 2010) |

Andrieux,L.O. *et al.* (2007) GATA-1 Is Essential in EGF-Mediated Induction of Nucleotide Excision Repair Activity and ERCC1 Expression through ERK2 in Human Hepatoma Cells. *Cancer Res*, **67**, 2114–2123.

Bitzer,M. *et al.* (2016) Resminostat plus sorafenib as second-line therapy of advanced hepatocellular carcinoma – The SHELTER study. *Journal of Hepatology*, **65**, 280–288.

Cao,Y. *et al.* (2020) Hsa-circRNA-103809 Promotes Hepatocellular Carcinoma Development via MicroRNA-1270/PLAG1 Like Zinc Finger 2 Axis. *Dig Dis Sci*.

Ding,W. *et al.* (2018) MicroRNA-493 suppresses cell proliferation and invasion by targeting ZFX in human hepatocellular carcinoma. *CBM*, **22**, 427–434.

Fu,Q. *et al.* (2018) Primary tumor-derived exosomes facilitate metastasis by regulating adhesion of circulating tumor cells via SMAD3 in liver cancer. *Oncogene*, **37**, 6105–6118.

Gan,W.-J. *et al.* (2016) RARγ-induced E-cadherin downregulation promotes hepatocellular carcinoma invasion and metastasis. *J Exp Clin Cancer Res*, **35**, 164.

Huang,Z. *et al.* (2015) Sp1 cooperates with Sp3 to upregulate MALAT1 expression in human hepatocellular carcinoma. *Oncology Reports*, **34**, 2403–2412.

Kremer-Tal,S. *et al.* (2004) Frequent inactivation of the tumor suppressor Kruppel-like factor 6 (KLF6) in hepatocellular carcinoma. *Hepatology*, **40**, 1047–1052.

Luo,W. *et al.* (2016) MYC associated zinc finger protein promotes the invasion and metastasis of hepatocellular carcinoma by inducing epithelial mesenchymal transition. *Oncotarget*, **7**, 86420–86432.

Mžik,M. *et al.* (2016) Aberrant methylation of tumour suppressor genes WT1, GATA5 and PAX5 in hepatocellular carcinoma. *Clinical Chemistry and Laboratory Medicine (CCLM)*, **54**.

Sano,K. *et al.* (2003) Overexpression of retinoic acid receptor alpha in hepatocellular carcinoma. *Clin Cancer Res*, **9**, 3679–3683.

Sharma,B.K. *et al.* (2016) Inhibitor of differentiation 1 transcription factor promotes metabolic reprogramming in hepatocellular carcinoma cells. *FASEB j.*, **30**, 262–275.

Valentino,T. *et al.* (2013) PATZ1 interacts with p53 and regulates expression of p53-target genes enhancing apoptosis or cell survival based on the cellular context. *Cell Death Dis*, **4**, e963–e963.

Wang,J. *et al.* (2020) NFAT2 overexpression suppresses the malignancy of hepatocellular carcinoma through inducing Egr2 expression. *BMC Cancer*, **20**, 966.

Yang,W.-X. *et al.* (2019) CDK1, CCNB1, CDC20, BUB1, MAD2L1, MCM3, BUB1B, MCM2, and RFC4 May Be Potential Therapeutic Targets for Hepatocellular Carcinoma Using Integrated Bioinformatic Analysis. *BioMed Research International*, **2019**, 1–16.

Zeng,T. *et al.* (2017) LncRNA-AF113014 promotes the expression of Egr2 by interaction with miR-20a to inhibit proliferation of hepatocellular carcinoma cells. *PLoS ONE*, **12**, e0177843.

Zhang,Q. *et al.* (2018) miR-3928v is induced by HBx via NF-κB/EGR1 and contributes to hepatocellular carcinoma malignancy by down-regulating VDAC3. *J Exp Clin Cancer Res*, **37**, 14.

Zhang,X. *et al.* (2018) Sp1-regulated transcription of RasGRP1 promotes hepatocellular carcinoma (HCC) proliferation. *Liver Int*, **38**, 2006–2017.

Zheng,Y. *et al.* (2016) Genome-wide DNA methylation analysis identifies candidate epigenetic markers and drivers of hepatocellular carcinoma. *Brief Bioinform*, bbw094.

Zhu,Y. *et al.* (2020) Sp2 promotes invasion and metastasis of hepatocellular carcinoma by targeting TRIB3 protein. *Cancer Med*, **9**, 3592–3603.

Zhu,Y.-Z. *et al.* (2010) Hepatitis B virus X protein induces hypermethylation of p16 ^INK4A^ promoter via DNA methyltransferases in the early stage of HBV-associated hepatocarcinogenesis. *Journal of Viral Hepatitis*, **17**, 98–107.

Zou,R.-C. *et al.* (2019) Identification of metabolism-associated pathways and genes involved in male and female liver cancer patients. *J Theor Biol*, **480**, 218–228.

**Supplemental Figure 1**. Coverages of switching regions and accuracies of DISMIR under different switching region thresholds. (A) Coverages of switching regions changing with different switching region thresholds. The gray line denotes the average coverage of DMRs identified by CancerDetector. (B) AUCs of DISMIR trained by switching regions identified with different switching region thresholds. Each condition was performed for ten times with random partition of training and test samples.


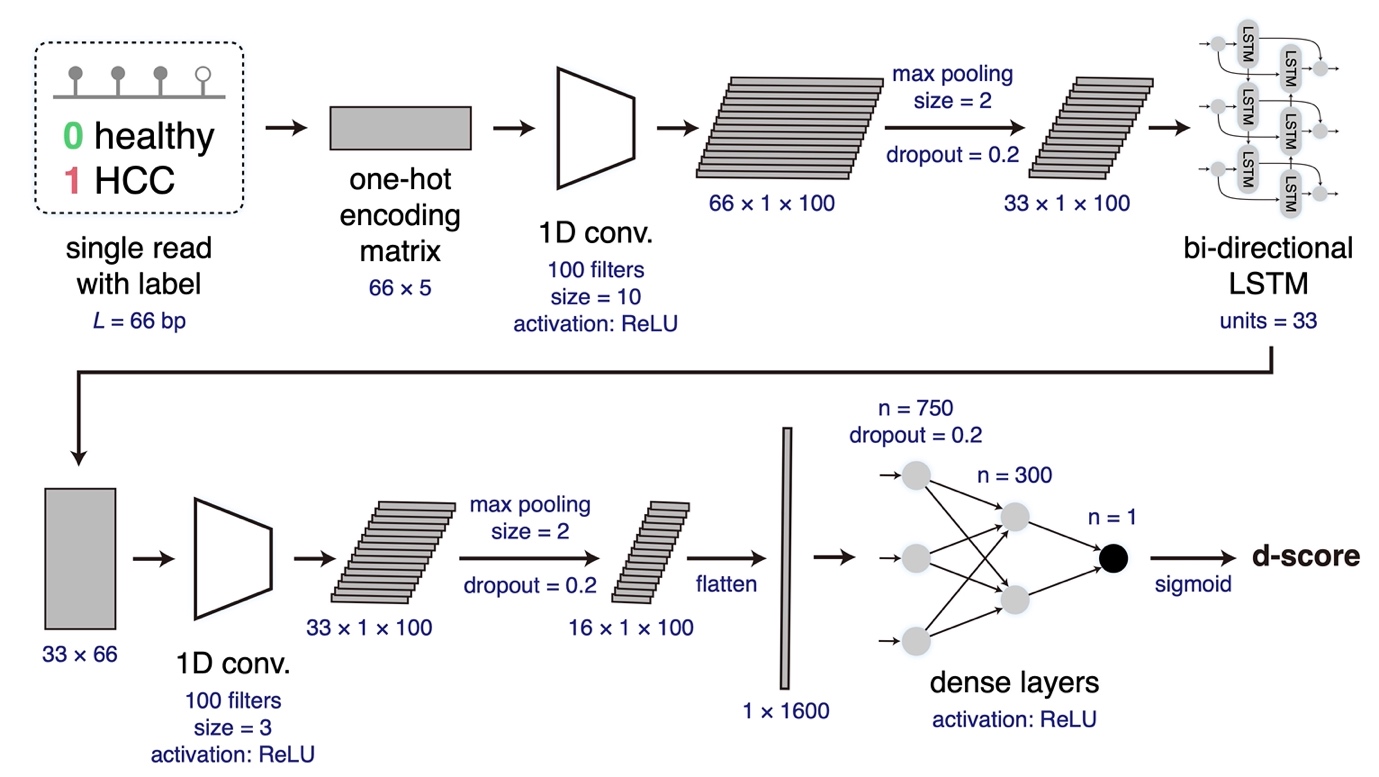


**Supplemental Figure 2**. Structure of the deep learning model.


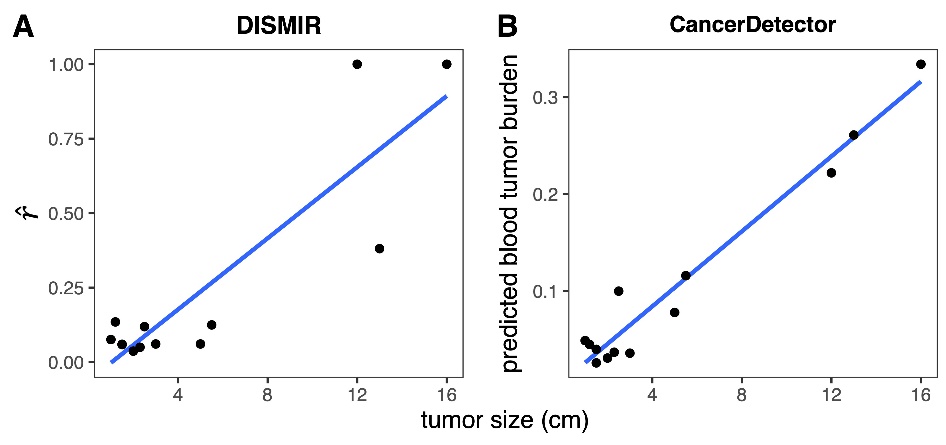


**Supplemental Figure 3**. The relationships between tumor sizes and predicted values of (A) DISMIR and (B) CancerDetector. Blue lines are the linear regression results of points. Here, CancerDetector denotes the method following the principle of the CancerDetector paper.

**Supplemental Figure 4**. Predicted results of DISMIR with hypo- and hyper-methylated switching regions. (A) ROC curves of DISMIR with hypo- and hyper-methylated switching regions. (B–C) Predicted values (*r*) of DISMIR with hypo- and hyper-methylated switching regions with (B) linear and (C) logarithmic scales.
